# Autistic traits affect interpersonal motor coordination by modulating strategic use of role-based behavior

**DOI:** 10.1186/s13229-017-0141-0

**Published:** 2017-06-09

**Authors:** Arianna Curioni, Ilaria Minio-Paluello, Lucia Maria Sacheli, Matteo Candidi, Salvatore Maria Aglioti

**Affiliations:** 1 Department of Psychology, Sapienza University of Rome, Via dei Marsi 78, Rome, Italy; 2 IRCCS Fondazione Santa Lucia, Via Ardeatina 306, Rome, Italy; 3 Department of Psychology and Milan Center for Neuroscience (NeuroMi), Bicocca University of Milan, Piazza dell’ Ateneo Nuovo 1, Milan, Italy; 4 Department of Cognitive Science, Central European University, October 6 Street, Budapest, Hungary

**Keywords:** Autism, Joint actions, Interpersonal coordination, Coordination strategies, Role taking, Cooperation

## Abstract

**Background:**

Despite the fact that deficits in social communication and interaction are at the core of Autism Spectrum Conditions (ASC), no study has yet tested individuals on a continuum from neurotypical development to autism in an on-line, cooperative, joint action task. In our study, we aimed to assess whether the degree of autistic traits affects participants' ability to modulate their motor behavior while interacting in a Joint Grasping task and according to their given role.

**Methods:**

Sixteen pairs of adult participants played a cooperative social interactive game in which they had to synchronize their reach-to-grasp movements. Pairs were comprised of one ASC and one neurotypical with no cognitive disability. In alternate experimental blocks, one participant knew what action to perform (instructed role) while the other had to infer it from his/her partner’s action (adaptive role). When in the adaptive condition, participants were told to respond with an action that was either opposite or similar to their partner. Participants also played a non-social control game in which they had to synchronize with a non-biological stimulus.

**Results:**

In the social interactive task, higher degree of autistic traits predicted less ability to modulate joint action according to one’s interactive role. In the non-social task, autistic traits did not predict differences in movement preparation and planning, thus ruling out the possibility that social interactive task results were due to basic motor or executive function difficulties. Furthermore, when participants played the non-social game, the higher their autistic traits, the more they were interfered by the non-biological stimulus.

**Conclusions:**

Our study shows for the first time that high autistic traits predict a stereotypical interaction style when individuals are required to modulate their movements in order to coordinate with their partner according to their role in a joint action task. Specifically, the infrequent emergence of role-based motor behavior modulation during on-line motor cooperation in participants with high autistic traits sheds light on the numerous difficulties ASC have in nonverbal social interactions.

**Electronic supplementary material:**

The online version of this article (doi:10.1186/s13229-017-0141-0) contains supplementary material, which is available to authorized users.

## Background

Autism Spectrum Conditions (ASC) are characterized by such deficits in social communication and interaction as the difficulty in understanding another person’s perspective, reduced use of verbal and nonverbal signals to regulate interaction, reduced use and understanding of gestures, abnormalities in body language, and difficulties in reciprocal, cooperative social play [1].

ASC cooperative behavior has usually been investigated in one of two ways: (1) via high-order cognitive tasks requiring participants to make a decision after having inferred something about their partner’s choices, as in computer-based social hunting games [2] or the prisoner’s dilemma [3–5]; or (2) via motor tasks requiring participants to take turns, foresee their partner’s actions and possibly modify their behavior, e.g., passing an object while taking the partner’s starting comfort state into account [6, 7].

Very few studies have tested ASC participants in on-line cooperative motor tasks in which they have to coordinate their actions with those of a partner to achieve a common goal. Distinctive features of realistic interpersonal coordination include the need to predict, imitate, or complement what a partner will do [8, 9], as well as the need to signal one’s intentions to the other by modulating movements’ kinematics [10–15]. These processes are managed according to contextual conditions such as, for example, who is the leader of an interaction and who is the follower required to adapt [16–20].

Coordination strategies to achieve successful interactions are likely based on a variety of motor, social, and cognitive skills. In particular, it has been proposed that on-line prediction of and adaptation to another person’s action may rely on a sensory-motor simulation of the observed action [21–24] that is integrated into one’s own action plan [25–28]. On-line, reciprocal time- and space-coordination represents a strong test case for social skills, as it requires predictive and sensorimotor monitoring processes to be coupled with higher order social functions (e.g., joint attention, role taking, theory of mind, visual perspective taking) that are impaired early on in the development of ASC individuals [29].

Some studies investigating on-line motor coordination in ASC showed that ASC children are less able than children with developmental delay to coordinate their actions when completing simple motor interactive tasks that require two persons in cooperation to make an object/toy function [30, 31]. Other research has employed a dynamical approach to investigate social synchronization performances in children and adolescents with ASC. Using a paradigm already tested with adult participants [32], Marsh [33] measured the amount of spontaneous interpersonal synchronization that occurred between children with ASC and their parents while sitting on rocking chairs and compared their findings to dyads of parents-children with typical development. Their results indicate that children with ASC showed less entrainment with their parents compared to controls. Similarly, in a study that used a pendulum coordination paradigm, it was shown that children and adolescents with ASC engage in less synchronized behavior than controls, specifically in the anti-phase condition [34]. Taken together, these studies suggest that low-level attentional and motor deficits in individuals with ASC may prevent them from experiencing connectedness with others through spontaneous synchronization. Such synchronization may be a first step toward the ability to coordinate and cooperate with others. Stoit and colleagues [35] measured motor performance while two participants (either two ASC or two control children or adolescents) worked together to lift a virtual bar without dropping the virtual ball placed on top of it. Participants could either use a single joystick to control the movement of one side of the bar, or two joysticks to control both sides. Results showed that ASC participants did not slow their reaction times in movement onset to accommodate their partner. They also favored the single joystick condition for its stronger sense of agency compared to the two joystick condition, and they were less able to synchronize their responses, causing the ball to drop shortly after movement onset. Despite the use of joysticks in their task, which reduces the impact of sensory-motor simulation, Stoit and colleagues [36] interpreted their results as evidence for the reduced ability of ASC participants to predict and coordinate their actions with a partner. These findings are in line with impaired internal modeling in ASC [37].

Importantly, no study has tested whether autistic traits impact sensory-motor simulation used to predict a partner’s behavior and adapt to it, or the ability to modulate one’s own motor behavior depending on the role played in the interaction. Our study investigates the motor cooperative behavior of ASC and neurotypical individuals during an on-line joint action task in order to address these questions for the first time.

We applied our cooperative joint action task (social interactive task [18]) by asking pairs of ASC and neurotypical participants to coordinate in space and time in order to synchronously grasp a bottle. Participants could either have a leader role, in which they were told what specific movement to perform toward the bottle (instructed condition) or a follower role, which required on-line adaption to their partner and the performance of same/opposite movements (adaptive condition). Our aim was to test whether autistic traits modulate (i) participants’ ability to adjust their behavior depending on their interactional role and (ii) sensorimotor simulation tested by comparing same and opposite movements. We predicted that participants, depending on their role in the interaction and their degree of autistic traits, would differently recruit prediction and adaptation strategies in order to ease motor cooperation and facilitate on-line interpersonal coordination. To distinguish between social interactive performance and baseline level of motor, perceptual, and executive abilities, we used a newly developed non-social task in which participants had to coordinate their reach-to-grasp movements with a non-biological stimulus.

### Participants

We tested 19 pairs of same sex, adult participants (Table 1). Each pair consisted of one neurotypical (NT) participant and one ASC participant without cognitive disability. All participants took part in both experiments (i.e., social interactive task and non-social task).

**Table 1:** Participants' demographic information and symptoms' severity

	ASC *N* = 16	NT *N* = 16	*t* test
Sex ratio female:male	3:13	3:13	–
Mean age (age range)	26.1 (18–42)	29.6 (17–49)	*t*(30) = −1.11, *p* = 0.27
Mean Autism Quotient	31.9 (6.7)	10.3 (5.3)	*t*(30) = 10.2, *p* < 0.001
ADOS Social Interaction and Communication	12 (5–17)	–	–

Participants’ cognitive ability was assessed by either the Raven’s Standard Progressive Matrices test (RSPM) [38] or the Italian version of the Wechsler Adult Intelligence Scale Revised (WAIS-R) [39] (see Additional file 1: Table S2 for individual scores). All participants had an IQ within the normal range (RSPM ASC: *N* = 9, mean = 97 range = 71–100; NT: *N* = 9, mean = 94.5 range = 79–100; WAIS ASC: *N* = 5, average full-scale IQ = 88.8, range = 75–98). IQ scores were not available for one NT and two ASC participants. As these participants had all received a master’s degree, we considered them to be within the normal range. ASC participants received an Asperger syndrome diagnosis according to DSM IV criteria [1] and completed the Autism Diagnostic Interview-Revised [34] and/or the Autism Diagnostic Observation Schedule-Generic [40] (see Additional file 1: Table S1). It was not possible to run the ADI-R with the parents of 5 ASC participants. Before the experiment, all participants completed the Autism Quotient questionnaire, which measures the number of autistic traits in adults [41].

Two pairs were excluded from analyses as they showed outlier kinematic or behavioral values with respect to the group mean; one pair was excluded due to data loss. Our final sample consisted of 32 participants (Table 1). Both females (*N* = 6) and males (*N* = 26) were included for recruitment convenience.

In nine of the pairs, participants already knew each other, while participants in the seven other pairs were informed about their diagnostic status and familiarized with each other for 1 h before the experiment began.

The study was approved by the local ethics committee at IRCCS Fondazione Santa Lucia and conducted in accordance with the Declaration of Helsinki. Participants gave informed consent prior to participation in the study and were paid for their time.

## Methods

### Experiment 1: social interactive task

In experiment 1, we aimed to test individual motor behavior and pair coordination in a joint cooperative task [18] in which participants were asked to grasp a bottle-shaped object placed in front of them (one for each participant) as synchronously as possible with their partner. The experiment lasted approximately 1.5 h. Participants were either told where to grasp the bottle (instructed condition) or required to on-line adapt to their partner’s movement (adaptive condition). Fifty percent of the time, participants were asked to perform movements opposite to those of their partner (opposite vs. same).

This task allowed us to measure whether autistic traits modulate (i) participants’ ability to strategically change behavior in the adaptive vs. instructed condition or (ii) recruitment of on-line sensory-motor simulation when performing same vs. opposite movements.

#### Exp. 1 ﻿Methods Exp. 1 setup

**Fig. 1:**
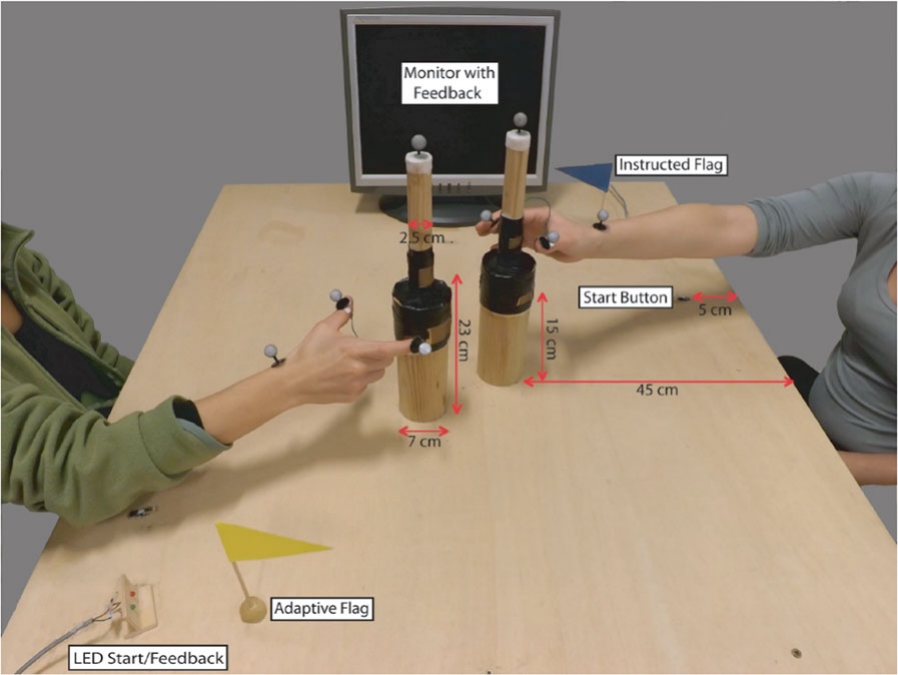
Setup of the social interactive task. Here, participants perform an opposite trial. The participant on the right is going to grasp the upper part of her bottle-shaped object via a precision grip while the participant on the left is going to grasp the lower part of her bottle-shaped object via a power grip

Participants sat at either side of a table equipped with start buttons, bottle-shaped objects, go/feedback LEDs, and a monitor to provide task instructions and performance feedback after each trial (Fig. 1).

The bottom part of the bottle had to be grasped with a power grip while the top part had to be grasped with a precision grip. Grasp time was recorded by two copper plates placed at the bottom and top of the bottle.

#### Exp. 1 procedure

In each block participants took one of the following two roles in counterbalanced order:

- Instructed: participants were told where to grasp their object before starting their movement. Through headphones, they heard either the word “down” (i.e., grasp the bottom part of the bottle via a power grip) or “up” (i.e., grasp the top part of the bottle via a precision grip). Participants had to on-line *adapt in time* to their partner (i.e., no need to on-line change the target of their movement) in order to correctly perform the task and maximize interpersonal synchrony in touching the object.
- Adaptive: participants did not know where to grasp their object in advance, having to on-line select their movements based on the movement of their partner. Through headphones, they heard either the word “opposite” (i.e., grasp the bottle in the opposite location your partner does) or “same” (i.e., grasp the bottle in the same location your partner does). Participants had to take their partner’s movement into account and on-line *adapt in both space and time* to it in order to correctly perform the task and maximize interpersonal synchrony in touching the object.

In each experimental block, participants knew which role they were to play by way of color-coded instructions displayed on the monitor at the beginning of each block, as well as a color-coded flag placed next to their start button throughout the entire block (Fig. 1).

##### Trial timeline

Participants heard their instructions via headphones. They could start their reach-to-grasp movement when a pair of LEDs placed near the right hand of their partner turned from red to green.

Asynchrony between participants’ grasps was calculated as the absolute time interval between the two participants grasping times on their bottle-shaped objects (grasping asynchrony, see the “Exp 1 data processing” section). Performance feedback was provided via LEDs (green = win, red = loss) and displayed on the monitor (i.e., “1 point” or “0 point” and the amount of asynchrony in milliseconds). To encourage participants’ commitment during the task, monetary reward depended on points collected and average level of synchrony.

Pairs earned a point when their asynchrony in grasping the objects was smaller than a variable time window tailored to pair performance via a staircase procedure. This caused the task to become harder as participants improved in their coordination performance, allowing us to control for task difficulty and learning effects.

The experimental procedure was controlled by an E-Prime script that randomized conditions, and allowed us to on-line record start time, grasp time, grasp location, as well as to provide auditory instructions and performance feedback.

##### Task structure

Pairs performed 4 adaptive/instructed blocks of 32 trials each (128 trials in total, 16 per condition). Role (instructed/adaptive) was fixed within blocks and counterbalanced between participants, while congruency (opposite/same) and position of the target (down/up) were pseudo-randomized within block in order to avoid more than 4 consecutive opposite/same and 2 down/up trials. Experimental design was therefore a 2 role (instructed/adaptive) × 2 congruency (opposite/same) × 2 position (down/up) within-subject design.

#### Exp. 1 data processing

Only correct trials were entered in the analysis (i.e., incorrect trials, in which at least one of the participants performed a wrong movement, were used to estimate pair accuracy; see below). Trials were discarded when at least one participant in the pair started his/her movement before the instructions, or if one of the grasps was not effective in closing the circuit, thus preventing touch time collection (mean % per pair = 17, SD = 9, range = 6–34%).

##### Pair performance

For each trial, we considered the following behavioral measures of pair performance:

- Pair accuracy (ACC): percentage of trials in which both participants grasped their object in the correct location.
- Grasping asynchrony (ms) (GraspAsynch): trial-by-trial absolute time interval between each participant’s grasp (hand contact) of the object [abs(participant1_grasp time − participant2_grasp time)].
- Difference in reaction times (ms) (∆RT): trial-by-trial time interval between the release of the start button by the participant in the adaptive role and that of the participant in the instructed role (**∆**RT = adaptive RT − instructed RT).

##### Individual performance

For each trial, we considered the following behavioral measures of individual performance:

- Reaction time (ms) (RT): interval between auditory instruction delivery time and start button release time.
- Movement duration (ms) (MD): interval between start button release time and grasp time.

For each dependent variable, participant, and condition, we excluded values that fell 2.5 standard deviations (s.d.) above or below the mean as outliers. Z-transformation within participant/couple and across conditions was performed on all dependent variables but **∆** *RT*, as it would not have allowed us to investigate trial-by-trial time course of pair interaction.

Movement’s spatial kinematics (max index-thumb aperture, max wrist height) was also recorded with a SMART-D motion capture system (Bioengineering Technology & Systems (B|T|S)). See Additional file 1 for methods and results.

#### Exp. 1 data analysis

In our social interactive task, we wanted to investigate behavioral changes outside the classic and often limiting case-control framework. When possible, we thus analyzed the data from all participants collapsed via an ANOVA with the number of autistic traits (AQ) as continuous predictor.

We report parametric repeated measures ANOVA when variables are all normally distributed (Shapiro-Wilk’s test) or when their skewness and kurtosis can be considered normal [42]. This is the case for grasping asynchrony, delta reaction time, and movement duration. For the latter variable, being it a measure of individual performance, the analysis also includes the number of autistic traits (AQ) as a moderator.

For reaction time, we report in the main text parametric ANOVA with AQ as a continuous predictor because (i) deviation from normality is minor, (ii) parametric analyses suggest a role for the AQ in mediating the effects, (iii) nonparametric results generally confirm nonparametric ones, and (iv) there is not a simple non-parametric counterpart of mixed model ANOVA nor of ANOVA with a continuous predictor (in our case, the AQ). We include nonparametric analyses in the Additional file 1.

Lastly, we report nonparametric analysis when the majority of variables are not normally distributed (Shapiro-Wilk’s test) and their skewness and kurtosis are not normal [42].

##### Pair performance

ACC entered nonparametric, repeated measure, Friedman ANOVA, and, if significant, we ran nonparametric Wilcoxon matched pair tests.

GraspAsynch and ∆RT entered separate repeated measures analyses of variance (ANOVAs) with 2 role (instructed/adaptive) × 2 congruency (opposite/same) as within-subjects factors. In pair performance analyses only, (i) position (down/up) was collapsed because 50% of trials (i.e., in the opposite condition) involve one participant going down while the other going up; and (ii) role was coded according to the role of the ASC participant, i.e., the trial was coded as adaptive when the ASC participant of the pair had the adaptive role.

##### Individual performance

RT and MD entered separate repeated measures ANOVAs with 2 role (instructed/adaptive) × 2 congruency (opposite/same) × 2 position (down/up) as within-subjects factors and the autism quotient [41] scores as moderator. The use of the AQ as a moderator in the analyses on individual performance allowed us to test all participants on a continuum and investigate the influence of autistic traits across the entire spectrum from neurotypical to neurodiverse behavior. The AQ moderator was centered across all subjects, i.e., individual AQ score − group mean AQ (21.06) [43].

As a control analysis, we also divided participants depending on their diagnosis and entered the same dependent variables in a mixed model ANOVA with 2 role (instructed/adaptive) × 2 congruency (opposite/same) × 2 position (down/up) as within-subjects factors and group (ASC/NT) as between subjects factor (see Additional file 1).

All tests of significance were based upon an α level of 0.05. Significant interactions and main effects were analyzed by Newman-Keuls post hoc tests. In order to make the role of the AQ continuous predictor easier to interpret, significant interactions including the AQ were further tested using a correlational approach. From each correlation, participants with absolute standard residuals greater than 2 (range −2.34–2.48) were removed as outliers.

## Results

### Exp. 1

#### Pair performance

Friedman ANOVA on **ACC** did not show significant main effects of role and congruency, nor a significant role × congruency significant interaction (chi square (16,3) = 3.22, p = 0.36), indicating that pairs reached an equal level of accuracy in all experimental conditions.

Analysis on GraspAsynch showed a significant main effect of congruency (*F*(1,15) = 6.84, *p* = 0.02, *η* ^2^ = 0.31), with greater asynchrony when participants performed opposite vs. same movements. All other *p*s > 0.17.

**Fig. 2:**
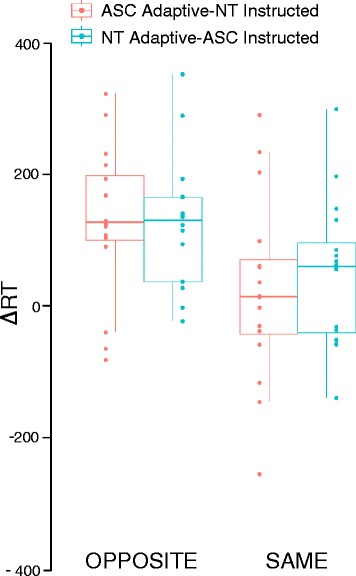
Difference in RT (*∆RT*) between participants playing in the same pair according to their interactional role and diagnostic status. *Lower and upper bars* correspond to the first and third quartiles respectively. *Horizontal dark lines* correspond to the median. ***p < .001, **p < .01

Analysis on **∆**RT showed a significant main effect of congruency (*F*(1,15) = 75.07, *p* < 0.00, *η* ^2^ = 0.83), indicating longer **∆**RT for opposite trials and a significant role × congruency interaction (*F*(1,15) = 5.27, *p* = 0.04, *η* ^2^ = 0.26) (Fig. 2). Note that positive **∆**RT values indicate that the participant in the adaptive role (either ASC or NT) started to move after his/her partner, while negative values indicate that he/she started to move before his/her partner. Post hoc tests showed that ∆RT values are longer in opposite vs. same trials (*p*s < 0.001) and that only in same trials there is a difference in ∆RT, depending on which pair member takes the adaptive role (*p* = 0.02). This difference is not significant in opposite trials (*p* = 0.60).

Mean ∆RT was always positive in opposite trials (significant one sample *t* tests against zero ASC adaptive *t*(16) = 4.75; NT adaptive t(16) = 5.16, *p*s < 0.001), meaning that the adaptive player always started to move after his/her partner, regardless of being ASC or NT. This was not the case for same trials, when ∆RT was positive only when NT had the adaptive role (one sample *t* tests against zero ASC *t*(16) = 0.92, *p* = 0.37; NT *t*(16) = 2.09, *p* = 0.05). That is, in same trials, ASC participants in the adaptive role started their movement at the same time as their partner, rather than waiting to see which grip their partner would make.

#### Individual performance

**Fig. 3:**
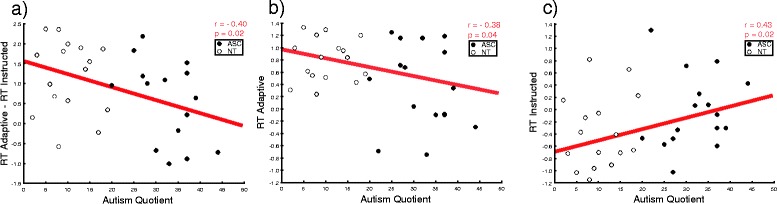
Correlations between individual RT and number of autistic traits (AQ) according to role in the social interaction. **a** The higher participant’s AQ, the smaller his/her RT difference for adaptive vs. instructed movements; **b** the higher participant’s AQ, the faster his/her RT for adaptive movements; **c** the higher participant’s AQ, the slower his/her RT for instructed movements

Analysis on RT showed a significant main effect of role (*F*(1,30) = 17.88, *p* < 0.001, *η* ^2^ = 0.37), indicating that participants in the adaptive role exhibited longer reaction times than those in the instructed role. This effect was modulated by participants’ autistic traits (role × AQ interaction = *F*(1,30) = 4.635, *p* = 0.039, *η* ^2^ = 0.13). To interpret this interaction, we subtracted individuals’ RTs in the instructed condition from their RTs in the adaptive condition (higher positive values index participants’ tendency to be slower when they need to adapt) and correlated this index with their AQ score. We found a negative correlation (*N* = 31, *r* = −0.40, *p* = 0.02), indicating that the lower the AQ, the longer the RT in the adaptive as compared to the instructed condition (i.e., indexing more strategic modulation of one’s RTs) (Fig. 3a).

Additionally, we investigated the correlation between individual mean RTs and the AQ moderator separately for the adaptive and instructed conditions. We found a negative linear correlation in the adaptive condition (*N* = 30, *r* = −0.38, *p* = 0.04), indicating that the higher the autistic traits, the shorter the time taken to initiate the movement (i.e., less strategic adaptation) (Fig. 3b). We also found a positive correlation in the instructed condition (*N* = 30, *r* = 0.43, *p* = 0.02), indicating that the higher the autistic traits, the longer the time taken to initiate the movement (Fig. 3c).

**Fig. 4:**
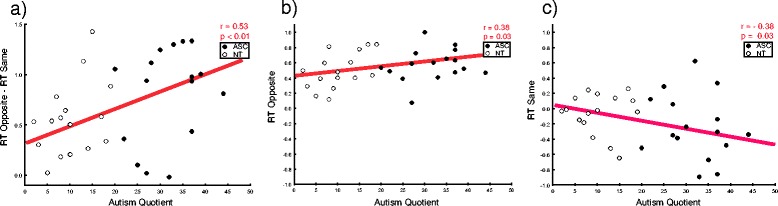
Correlations between individual RT and number of autistic traits (AQ) according to the congruency between observed and executed actions. **a** The higher participant’s AQ, the greater his/her RT difference for opposite vs. same movements; **b** the higher participant’s AQ, the slower his/her RT for opposite movements; **c** the higher participant’s AQ, the faster his/her RT for same movements

Furthermore, results also included significant main effects of position (*F*(1,30) = 42.78, *p* < 0.001, *η* ^2^ = 0.58) and congruency (*F*(1,30) = 90.84, *p* < 0.001, *η* ^2^ = 0.75). The former indicates that RTs were longer for up than they were for down grasps; the latter indicates that opposite movements caused longer RTs than same ones. This latter effect was modulated by the autistic traits of participants (congruency × AQ interaction *F*(1,30) = 5.8, *p* = 0.02, *η* ^2^ = 0.16). To interpret this interaction, we subtracted individuals’ RTs in the same condition from their RTs in the opposite condition (higher positive values index longer time to initiate the movement in opposite compared to same trials) and correlated this index with their AQ score. We found a positive correlation (*N* = 30, *r* = 0.53, *p* = 0.002), which indicates that the higher the AQ, the slower the RT in the opposite compared to the same condition (Fig. 4a). Additionally, we investigated the correlation between individual mean RTs and the AQ moderator separately for opposite and same trials. Correlational analysis showed that participants with higher autistic traits tended to show longer RT in opposite trials (*N* = 31, *r* = 0.38, *p* = 0.03) (Fig. 4b) and shorter RT in same trials (*N* = 31, *r* = −0.38, *p* = 0.03) (Fig. 4c).

Significant interactions include role × congruency (*F*(1,30) = 23.3, *p* < 0.001, *η* ^2^ = 0.43), congruency × position (*F*(1,30) = 31.7, *p* < 0.001, *η* ^2^ = 0.51), and role × congruency × position (*F*(1,30) = 22.0, *p* < 0.001, *η* ^2^ = 0.42). The latter indicates longer RT in opposite-up trials for participants in the adaptive condition (*p* = 0.001). All other *p*s > 0.16.

**Fig. 5:**
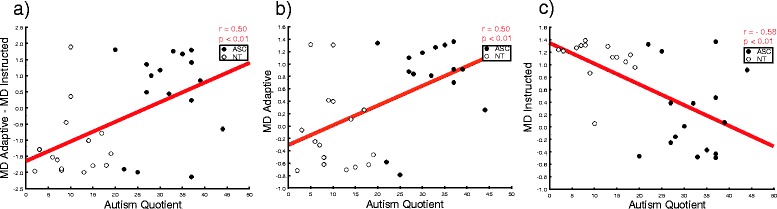
Correlations between individual movement duration (MD) and number of autistic traits (AQ) according to the role in the social interaction. **a** The higher participant’s AQ, the greater his/her MD difference for adaptive vs. instructed movements; **b** the higher participant’s AQ, the longer his/her MD for adaptive movements; **c** the higher participant’s AQ, the shorter his/her MD for instructed movements

Analysis on MD showed significant main effect of congruency (*F*(1,30) = 5.15, *p* = 0.03, *η* ^2^ = 0.14) and position (*F*(1,30) = 5.7, *p* = 0.02, *η* ^2^ = 0.16). The former indicates longer MD in opposite vs. same trials; the latter indicates longer MD in up vs. down trials (as expected given the setup physical constraints), as well as a significant role × AQ interaction (*F*(1,30) = 6.15, *p* = 0.02, *η* ^2^ = 0.17). To interpret the latter interaction, we subtracted individual MD in the instructed from individual MD in the adaptive condition (higher positive values index longer movement duration in participants playing adaptive compared to instructed) and correlated this index with the AQ. We found a positive correlation (*N* = 31, *r* = 0.50, *p* < 0.01). This indicates that the lower the AQ, the longer the MD in the instructed vs. the adaptive condition (Fig. 5a). The AQ moderator correlated negatively with MD in the instructed condition (*N* = 30, *r* = −0.58, *p* < 0.01) and positively in the adaptive condition (*N* = 31, *r* = 0.50, *p* < 0.01). This suggests that the lower the autistic traits in the instructed role, the longer the MD (Fig. 5b). The opposite pattern was true for the adaptive role (Fig. 5c).

Analysis on MD also showed a significant role × congruency interaction (*F*(1,30) = 36.3, *p* < 0.001, *η* ^2^ = 0.54) and a trend to significance of the role × congruency × AQ interaction (*F*(1,30) = 3.9, *p* = 0.06, η^2^ = 0.11). All other *ps* > 0.12. To interpret the latter interaction, we subtracted individual MD in the instructed condition from individual MD in the adaptive condition. We did this separately for opposite and same trials, correlating this index with the AQ. We found a positive correlation for both opposite and same trials (*N* = 31, *r* = 0.43, *p* < 0.02 and *N* = 31, *r* = 0.55, *p* < 0.001), indicating that the higher the AQ, the longer the MD in the adaptive vs. instructed condition of both trials. These results indicate that participants with high autistic traits performed slower movements in the adaptive condition, regardless of their congruency with their partner’s movement.

### Experiment 2: non-social task

**Fig. 6:**
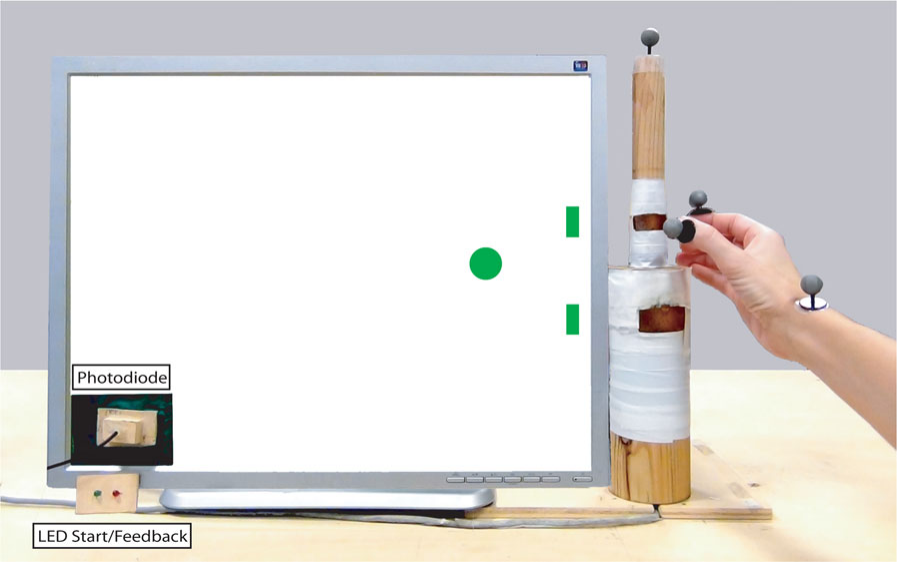
Setup of the non-social task. Example of a congruent trial in which the participant is about to grasp the upper part of the bottle-shaped object with a precision grip and the *dot* is about to stop next to the upper target

In experiment 2, we tested whether autistic traits modulate motor behavior and coordination abilities of ASC and NT participants in an individual coordination task. The task lasted approximately 1 h. As in the social task, participants had to grasp a bottle-shaped object in front of them, but this time they had to do so *in synchrony with* a dot (i.e., non-social stimulus) that moved on a screen toward two possible targets placed next to either the upper or lower part of the bottle (Fig. 6). Participants’ grasping location was either spatially congruent or incongruent with the dot’s final position on the screen. As in exp. 1, participants were told one of two things before each trial: (1) where to grasp the bottle (instructed condition), or (2) to grasp the bottle in the same/opposite position with respect to the dot, therefore having to on-line adapt to its trajectory (adaptive condition). We introduced the non-social task to control whether differences in motor performance and executive control per se (independently from the interactive context) were accountable for the results found in the social interactive task.

### Exp. 2 Methods

#### Exp. 2 setup

Participants sat in front of a 120 × 100 cm table with a LCD monitor used for stimuli presentation. Stimuli consisted of 128 videos of a dot moving along a diagonal straight line toward one of two green rectangles placed next to the grasping points on the bottle. The dot started its movement from a location spatially similar to the starting position of the partner's hand in exp. 1. In order to prevent participants from predicting the dot’s stopping time, its velocity profiles were taken from the kinematics of individuals’ wrists while reaching and grasping the bottles. Videos were created using MatLab software. The dot’s stopping times during the task were recorded by a photodiode attached to the screen and connected to a PC running E-Prime2 software via a TTL cable (Psychology Software Tools Inc., Pittsburgh, PA). Participants received auditory instructions as in exp. 1.

#### Exp. 2 procedure

As described in exp. 1, participants performed the task while following one of two different roles (instructed or adaptive, in counterbalanced order) and were asked to perform opposite or same movements with respect to the dot’s trajectory and target location.

Trial-time line and Task structure is same as exp. 1.

#### Exp. 2 data processing

Only correct trials were entered in the analysis. Trials in which participants started their movement before hearing the auditory instructions were discarded, as were those in which the wrong part of the bottle was grasped (mean % per participant = 9, SD = 5, range = 1–21) (or used to compute ACC, see below).

For each trial, we considered the following behavioral measures:

- Accuracy (ACC): percentage of trials in which participant grasped the object in the correct location.
- Grasping asynchrony (ms) (GraspAsynch): trial-by-trial absolute time interval between participant’s grasp time and dot’s stop time [abs(participant_grasp time − dot_stop time)].
- Reaction time (ms) (RT): interval between delivery time of auditory instructions and start button release time.
- Movement duration (ms) (MD): interval between start button release time and grasp time.

#### Exp. 2 data analysis

The data analysis of experiment 2 is as Exp. 1.

## Results

### Exp. 2

#### Individual performance

Friedman ANOVA on **ACC** was significant (chi square (32,7) = 16.55, *p* = 0.02). We then ran a Wilcoxon matched pair test in order to investigate differences in ACC between roles and between congruency. None of the contrasts survived Bonferroni’s correction for multiple comparisons (*p*s > 0.02, corrected *α* = 0.05/4 = 0.01), indicating that participants reached an equal level of accuracy in all experimental conditions.

Analysis on GraspAsynch showed significant main effects of role (*F*(1,30) = 55.19, *p* < 0.001, *η* ^2^ = 0.64) and position (*F*(1,30) = 16.14, *p* < 0.001, *η* ^2^ = 0.34). The former indicates that participants in the instructed role were more synchronous with the dot than in the adaptive role; the latter indicates that participants were more synchronous with the dot when grasping the lower part of the bottle. Results also included significant congruency × AQ (*F*(1,30) = 4.24, *p* = 0.048, *η* ^2^ = 0.12) and role × congruency (*F*(1,30) = 6.45, *p* = 0.016, *η* ^2^ = 0.17) interactions, explained by the significant third level Role x Congruency x AQ interaction (*F*(1,30) = 4.95, *p* = 0.03, *η* ^2^ = 0.14).

To interpret this last interaction, we calculated an index of the significant effect (the GraspAsynch difference between opposite and same trials, collapsed for the factor position as it did not interact with the others) for each role and correlated it with the AQ moderator (higher positive values indexing participants’ tendency to show worse GraspAsynch when performing opposite movements compared to same ones).

**Fig. 7:**
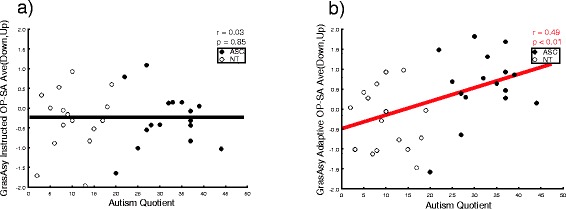
Correlations between individual grasping asynchrony (GraspAsynch) and number of autistic traits (AQ) according to movements’ congruency and participant’s diagnostic status. **a** No significant relationship between participants’ AQ and their GraspAsynch difference between opposite vs. same instructed movements, **b** the higher participant’s AQ, the larger his/her GraspAsynch difference for opposite vs. same adaptive movements

Correlational analysis revealed a positive linear correlation between AQ and adaptive (opposite-same) index (*N* = 31, *r* = 0.49, *p* = 0.005) where the higher the autistic traits, the worse the performance (i.e., greater asynchrony) in the opposite vs. the same condition (Fig. 7a). This suggests that participants with higher autistic traits were less able to ignore the dot’s spatially incongruent trajectory and target location in the adaptive role. Instead, the correlation between AQ and instructed (opposite-same) index was not significant (*N* = 31, *r* = 0.03, *p* = 0.85) (Fig. 7b). All other *p*s > 0.10.

Analysis on RT showed significant main effects of role (*F*(1,30) = 260.00, *p* < 0.001, *η* ^2^ = 0.90), and congruency (*F*(1,30) = 55.74, *p* < 0.001, *η* ^2^ = 0.65) that are explained by the significant second-order role × congruency interaction (*F*(1,30) = 36.61, *p* < 0.001, *η* ^2^ = 0.55). This indicates that participants showed longer RTs in opposite vs. same trials when taking the adaptive (*p* < 0.001) but not the instructed (*p* = 0.33) role. Results also show a significant role × position interaction (*F*(1,30) = 14.70, *p* < 0.001, *η* ^2^ = 0.32), indicating that RTs are longer for up trials when taking the instructed (*p* = 0.001) but not the adaptive (*p *= 0.07) role. The absence of significant interactions with the AQ moderator (all *p*s > 0.13) indicates that autistic traits did not modulate participants’ RTs in the individual coordination task. All other *p*s > 0.21.

Analysis on MD showed significant main effects of congruency (*F*(1,30) = 94.41, *p* < 0.001, *η* ^2^ = 0.75) and position (*F*(1,30) = 94.41, *p* < 0.001, *η* ^2^ = 0.75), as well as a significant congruency × position interaction (*F*(1,30) = 4.25, *p* = 0.048, *η* ^2^ = 0.12) that was explained by the significant higher order role × congruency × position × AQ interaction (*F*(1,30) = 6.07, *p* = 0.01, *η* ^2^ = 0.17). To interpret this last interaction, we calculated the difference between individuals’ mean MD in opposite minus same trials for each role and position, and correlated it with the AQ moderator. Correlational analysis revealed a positive linear correlation between AQ and the adaptive (opposite-same) down index (*N* = 32, *r* = 0.36, *p* = 0.04). This indicates that in the adaptive role, movement duration in the opposite down trials is longer the higher the autistic traits. This suggests that when performing a down movement in the adaptive role, participants with higher autistic traits were less able to ignore the dot’s upward spatially incongruent trajectory and target location. All other *p*s > 0.12.

## Discussion

The goal of the present study was to investigate cooperative motor behavior of participants on a continuum from neurotypical development to autism. Given the high variability within the autism spectrum and the often blurry distinction between neurotypical and neurodiverse behavior when considering ASC individuals without cognitive disability, we favored an investigation that would go beyond dichotomous diagnostic boundaries. Our study addressed if and how the number of autistic traits impacts participants’ ability to aid motor cooperation and facilitate on-line interpersonal coordination by predicting and adapting to the movements of a partner, as well as by adjusting one’s behavior depending on the role taken during the interaction.

### Social interactive task: the higher your autistic traits, the less you wait for your partner’s movement

Pairs of ASC and NT participants were less coordinated (i.e., synchronous) when they had to perform opposite movements, regardless of which pair member took the adaptive or instructed role. Previous results on pairs of NT participants [18, 44] instead showed no difference in coordination between same and opposite trials. Such an unexpected difference may be related to the fact that interaction is facilitated by within-group similarities in communication and interaction styles [45, 46], while interactions between out-group individuals may be more difficult [40]. Results on the difference in reaction times showed that when NT played adaptive, they always waited for their instructed partner to start his/her movement. This was not the case for ASC participants, who did not wait for their partner when performing same movements. A possible interpretation is that ASC participants may not feel the need to gather additional information from their partner’s movement in same trials and therefore start to move independently from him/her as soon as they hear the instructions. This is supported by individual reaction time results from the adaptive condition showing that the higher the autistic traits, the faster participants started to move.

### Social interactive task: the higher your autistic traits, the less you modulate your movement duration according to your role

Modulation of action planning and execution during joint action depends on the role individuals take to achieve the shared goal [19, 47]. In our task, successful coordination required participants not only to represent their individual task (e.g., grasp the bottom part of the bottle) but also to integrate it with their partner’s one (i.e., guide the interaction when playing instructed or adapt to their partner when playing adaptive). Our results show that in the instructed condition, participants took more time to perform movements than in the adaptive condition and that this was more true for participants with low autistic traits.

A number of studies have found that ASC participants have longer reaction times and movement durations than controls in simple goal-directed reach-to-grasp movements. These results have been interpreted either as atypical movement planning [48–52] or atypical action execution [53].

Our findings seem unlikely to be related to either movement planning or execution deficits per se, as the differences in motor behavior are selectively modulated by the role taken in the interaction. We interpret them in the light of previous studies on sensorimotor communication strategies during joint action, which found that neurotypical participants engage in systematic modulations of their movements to foster coordination and synchrony [10, 13, 15, 18, 19, 26].

When taking the instructed role, our participants with low autistic traits may have therefore slowed their movement in an attempt to reach better pair synchronization by making adaptation easier for their partner. In the adaptive condition, on the other hand, participants with low autistic traits showed shorter movement duration, possibly because they had to keep up with their high autistic traits partner who did not slow his/her movement to foster coordination. ASC reduced use of communicative and adaptive strategies to facilitate coordination during joint action suggests a difficulty in on-line updating individual actions according to the constraints of the interactive task, which might relate to the ASC tendency to not benefit from an interactive context, e.g., when they have to detect an interacting agent presented via point light display [54]. On the one hand, this might be linked to the ASC difficulty in picking up subtle, interactive-related cues from another person’s kinematics, and to ASC reduced use of such cues during an interaction. On the other hand, previous studies have shown that ASC children are able to cognitively represent another person’s task, as well as his/her relative needs [30], and that ASC adults are even interfered by it when playing next to another individual [55]. This may suggest that the absence of role-based adjustment of motor behavior in participants with high autistic traits might not be due to a lack of understanding that one’s partner has a different role, but rather to a reduced ability in recruiting strategies aimed to facilitate the other’s task [13].

### Non-social task: the higher your autistic traits, the more you are interfered by a non-social stimulus

The results of our non-social reach-to-grasp task are informative about individuals’ motor performance in a way that goes beyond the social nature of the interactive task and support the idea that the social task’s results do not depend on AQ-related differences in motor planning or execution. Coordination performance in the adaptive-opposite condition was predicted by participants’ autistic traits, i.e., the higher the autistic traits, the worse participants were able to synchronize with the dot when they had to move toward an opposite location. Worse performance in opposite vs. same trials can be considered as evidence of interference effect exerted by the incongruent trajectory of the observed dot over participants’ movement execution. Previous studies found no difference in the interference effect when NT and ASC participants observe real human motion or a dot moving with a biological velocity profile [56, 57]. Further, no group difference was found in classical response compatibility tasks where the observed movement is irrelevant [58–60]. Accordingly, autistic traits in our task did not predict performance in the instructed condition, where no on-line coordination in space was required. On the contrary, autistic traits modulated the interference effect exerted by the dot in the adaptive condition, when participants needed to take the dot’s trajectory into account in order to on-line adapt to it and select the target location. Given the significant interaction between role (instructed/adaptive) and congruency (same/opposite), we interpret our results as evidence of ASC difficulty in both ignoring the dot’s opposite trajectory and target location and inhibiting the automatic tendency to follow a similar trajectory, which might relate to ASC-reduced executive control [61–63]. Consistent with our results on synchrony performance, participants with higher autistic traits showed longer movement duration in the adaptive-opposite condition, which could be regarded as another index of interference effect. Due to the setup’s physical properties, this interference effect might only become apparent when participants follow the dot and go up, while all that is required is to go down and grasp the bottom part of the object.

Motor difficulties, often present in ASC individuals [64, 52], could contribute to the AQ-related differences found in the current experiments. The reach-to-grasp movement has been studied in autism [65] and a recent review of ASC reach-to-grasp [66] reports that ASC individuals show longer movement duration ([67, 68] but see [36]), greater change in acceleration ([68], but see [65]), and slower reaction times ([48] but see [67]) than neurotypicals. In our non-social task, the fact that the number of autistic traits’ effect on movement duration and synchrony is always based on the role played by participants seems to suggest that these effects are not related to action execution per se. In addition, the absence of an interaction with participants’ autistic traits in the analyses on reaction times clarifies that, in a non-social context in which participants had to coordinate their reach-to-grasp movements with a non-biological stimulus, autistic traits do not predict differences in movement preparation and planning. Therefore, basic differences in motor behavior appear not to be responsible for the results of the social interactive task.

## Conclusions

In this study, we investigated for the first time the influence of autistic traits on the ability to engage in a cooperative joint action task requiring predictive and adaptive abilities. We tested pairs of ASC and neurotypical participants in a joint-grasping task in which they had to synchronously grasp objects in front of them. By assigning participants to the instructed or adaptive role, we were able to investigate their ability to modify their behavior consistently with their role in order to ease coordination. We also tested participants in a non-social coordination task that allowed us to control for differences in motor execution per se. Results showed that higher autistic traits lessened participants’ ability to modulate their joint action according to their interactive role in the social task. In particular, participants with high autistic traits did not wait for their partner and showed longer movement duration when performing both same and opposite actions during trials in which they needed to adapt to their partner’s movement. No such effect was found in the conditions that did not require adaptation to the partner’s movements nor was it found in the non-social task. Indeed, autistic traits did not predict differences in movement preparation and planning in the non-social task, thus ruling out the possibility that the results of the social interactive task were due to basic motor or executive function difficulties. Overall, reduced use of communicative and adaptive strategies to facilitate coordination during joint action seems to suggest that participants with high autistic traits might find difficult to on-line update their action plans according to the constraints of the interactive task. Such reduced interpersonal coordination strategies during on-line motor cooperation may shed light on various ASC difficulties in social interaction.

### Study limitations

We should be cautious to generalize our results to the whole ASC population, as we only tested ASC individuals with no cognitive disability and, even if our sample size is consistent with that of similar published studies [18, 26], it consisted of a relatively small ASC group (*N* = 16) that may not represent ASC heterogeneity.

Having one ASC and one NT participant cooperating in the joint action task allowed us to investigate, for the first time, interactive dynamics when participants with different levels of social and communicative skills interact, as is often the case in real life. The present study did not also test the same NT participants playing with another NT. This condition would allow for the investigation of an additional experimental question, that is, whether NT participants change their motor behavior when they interact with ASC compared to a NT partner. We might expect NT participants to be more interfered by NT than ASC motor behavior, as it is more similar to their own and they might consider it more interpretable, informative, and reliable as a result [64]. Future studies could clarify this issue.

## Additional file

Additional file 1: Table S1. Individual ADOS and ADI scores. **Table S2.** Individual Intelligence scales scores. (DOCX 46.5 kb)

## Abbreviations

∆RT: Difference in reaction times
ASC: Autism Spectrum Conditions
GraspAsynch: Grasping asynchrony
MD: Movement duration
NT: Neurotypicals
RT: Reaction times

## References

[1] American Psychiatric Association (1994). Diagnostic and statistical manual of mental disorders, fourth edition (DSM-IV).

[2] Yoshida W, Dziobek I, Kliemann D, Heekeren HR, Friston KJ, Dolan RJ (2010). Cooperation and heterogeneity of the autistic mind. J Neurosci.

[3] Downs A, Smith T (2004). Emotional understanding, cooperation, and social behavior in high-functioning children with autism. J Autism Dev Disord.

[4] Li J, Zhu L, Gummerum M (2014). The relationship between moral judgment and cooperation in children with high-functioning autism. Sci Rep.

[5] Edmiston EK, Merkle K, Corbett BA (2015). Neural and cortisol responses during play with human and computer partners in children with autism. Soc Cogn Affect Neurosci.

[6] Gonzalez DA, Glazebrook CM, Studenka B, Lyons J (2013). Motor interactions with another person: do individuals with autism spectrum disorder plan ahead?. Front Integr Neurosci.

[7] Fitzpatrick P, Diorio R, Richardson M, Schmidt R (2013). Dynamical methods for evaluating the time-dependent unfolding of social coordination in children with autism. Front Integr Neurosci.

[8] Knoblich G, Jordan JS (2003). Action coordination in groups and individuals: learning anticipatory control. J Exp Psychol.

[9] Sebanz N, Bekkering H, Knoblich G (2006). Joint action: bodies and minds moving together. Trends Cogn Sci.

[10] Pezzulo G, Dindo H (2011). What should I do next? Using shared representations to solve interaction problems. Exp Br Res.

[11] Candidi M, Curioni A, Donnarumma F, Sacheli LM, Pezzulo G (2015). Interactional leader–follower sensorimotor communication strategies during repetitive joint actions. J R Soc Interface.

[12] Sartori L, Becchio C, Bara BG, Castiello U (2009). Does the intention to communicate affect action kinematics?. Conscious Cogn.

[13] Vesper C, van der Wel RP, Knoblich G, Sebanz N (2011). Making oneself predictable: Reduced temporal variability facilitates joint action coordination. Exp Br Res.

[14] Vesper C, van der Wel RP, Knoblich G, Sebanz N (2013). Are you ready to jump? Predictive mechanisms in interpersonal coordination. J Exp Psychol Hum Percept Perform.

[15] Vesper C, Schmitz L, Safra L, Sebanz N, Knoblich G (2016). The role of shared visual information for joint action coordination. Cognition.

[16] Konvalinka I, Vuust P, Roepstorff A, Frith CD (2010). Follow you, follow me: continuous mutual prediction and adaptation in joint tapping. Q J Exp Psychol A.

[17] Noy L, Dekel E, Alon U (2011). The mirror game as a paradigm for studying the dynamics of two people improvising motion together. Proc Natl Acad Sci U S A.

[18] Sacheli LM, Tidoni E, Pavone EF, Aglioti SM, Candidi M (2013). Kinematics fingerprints of leader and follower role-taking during cooperative joint actions. Exp Br Res.

[19] Vesper C, Richardson MJ (2014). Strategic communication and behavioral coupling in asymmetric joint action. Exp Brain Res.

[20] Skewes JC, Skewes L, Michael J, Konvalinka I (2015). Synchronised and complementary coordination mechanisms in an asymmetric joint aiming task. Exp Br Res.

[21] Aglioti SM, Cesari P, Romani M, Urgesi C (2008). Action anticipation and motor resonance in elite basketball players. Nat Neurosci.

[22] Keysers C, Gazzola V (2014). Hebbian learning and predictive mirror neurons for actions, sensations and emotions. Phil Trans R Soc B Biol Sci.

[23] Makris S, Urgesi C (2015). Neural underpinnings of superior action prediction abilities in soccer players. Soc Cogn Affect Neurosci.

[24] Hadley LV, Novembre G, Keller PE, Pickering MJ (2015). Causal role of motor simulation in turn-taking behavior. J Neurosci.

[25] Kokal I, Gazzola V, Keysers C (2009). Acting together in and beyond the mirror neuron system. Neuroimage.

[26] Candidi M, Sacheli LM, Aglioti SM (2015). From muscles synergies and individual goals to interpersonal synergies and shared goals: mirror neurons and interpersonal action hierarchies: comment on “Grasping synergies: a motor-control approach to the mirror neuron mechanism” by D’Ausilio et al. Phys Life Rev.

[27] Sacheli LM, Aglioti SM, Candidi M (2015). Social cues to joint actions: the role of shared goals. Front Psychol.

[28] Sacheli LM, Candidi M, Era V, Aglioti SM (2015). Causative role of left aIPS in coding shared goals during human-avatar complementary joint actions. Nat Comm.

[29] Happé F, Frith U (2014). Annual research review: towards a developmental neuroscience of atypical social cognition. J Child Psychol Psychiatry.

[30] Liebal K, Colombi C, Rogers SJ, Warneken F, Tomasello M (2014). Helping and cooperation in children with autism. J Autism Dev Disord.

[31] Colombi C, Liebal K, Tomasello M, Young G, Warneken F, Rogers SJ (2009). Examining correlates of cooperation in autism imitation, joint attention, and understanding intentions. Autism.

[32] Richardson MJ, Marsh KL, Isenhower RW, Goodman JR, Schmidt RC (2007). Rocking together: dynamics of intentional and unintentional interpersonal coordination. Hum Mov Sci.

[33] Marsh KL, Isenhower RW, Richardson MJ, Helt M, Verbalis AD, Schmidt RC, Fein D (2013). Autism and social disconnection in interpersonal rocking. Front Integr Neurosci.

[34] Fitzpatrick P, Frazier JA, Cochran DM, Mitchell T, Coleman C, Schmidt RC. Impairments of social motor synchrony evident in autism spectrum disorder. Front Psychol. 2016;7. 10.3389/fpsyg.2016.01323 PMC5005316 27630599

[35] Stoit AM, van Schie HT, Riem M, Meulenbroek RG, Newman-Norlund RD, Slaats-Willemse DI (2011). Internal model deficits impair joint action in children and adolescents with autism spectrum disorders. Res Autism Spectr Disord.

[36] Cattaneo L, Fabbri-Destro M, Boria S, Pieraccini C, Monti A, Cossu G, Rizzolatti G (2007). Impairment of actions chains in autism and its possible role in intention understanding. Proc Natl Acad Sci U S A.

[37] Lord C, Rutter M, Le Couteur A (1994). Autism Diagnostic Interview-Revised: a revised version of a diagnostic interview for caregivers of individuals with possible pervasive developmental disorders. J Autism Dev Disord.

[38] Raven JC, Court JH, Raven J (1992). Standard progressive matrices.

[39] Wechsler D (1981). Manual for the Wechsler Adult Intelligence Scale–Revised.

[40] Lord C, Risi S, Lambrecht L, Cook EH, Leventhal BL, DiLavore PC, Pickles A, Rutter M (2000). The Autism Diagnostic Observation Schedule—Generic: a standard measure of social and communication deficits associated with the spectrum of autism. J Autism Dev Disord.

[41] Baron-Cohen S, Wheelwright S, Skinner R, Martin J, Clubley E (2001). The autism-spectrum quotient (AQ): evidence from asperger syndrome/hih-functioning autism, malesand females, scientists and mathematicians. J Autism Dev Disord.

[42] Field A. Discovering statistics using SPSS. London: SAGE; 2009.

[43] Schneider BA, Avivi-Reich M, Mozuraitis M (2015). A cautionary note on the use of the analysis of covariance (ANCOVA) in classification designs with and without within-subject factors. Front Psychol.

[44] Sacheli LM, Candidi M, Pavone EF, Tidoni E, Aglioti SM (2012). And yet they act together: interpersonal perception modulates visuo-motor interference and mutual adjustments during a joint-grasping task. PLoS One.

[45] Cook J (2016). From movement kinematics to social cognition: the case of autism. Phil Trans R Soc B Biol Sci.

[46] Edey R, Cook J, Brewer R, Johnson MH, Bird G, Press C (2016). Interaction takes two: typical adults exhibit mind-blindness towards those with Autism Spectrum Disorder. J Abnorm Psychol.

[47] Knoblich G, Butterfill S, Sebanz N, Ross B (2011). Psychological research on joint action: theory and data. The Psychology of Learning and Motivation.

[48] Glazebrook CM, Elliott D, Szatmari P (2008). How do individuals with autism plan their movements?. J Autism Dev Disord.

[49] Glazebrook C, Gonzalez D, Hansen S, Elliott D (2009). The role of vision for online control of manual aiming movements in persons with autism spectrum disorders. Autism.

[50] Rinehart NJ, Bellgrove MA, Tonge BJ, Brereton AV, Howells-Rankin D, Bradshaw JL (2006). An examination of movement kinematics in young people with high-functioning autism and Asperger’s disorder: further evidence for a motor planning deficit. J Autism Dev Disord.

[51] Fournier KA, Hass CJ, Naik SK, Lodha N, Cauraugh JH (2010). Motor coordination in autism spectrum disorders: a synthesis and meta-analysis. J Autism Dev Disord.

[52] Gowen E, Hamilton A (2013). Motor abilities in autism: a review using a computational context. J Autism Dev Disord.

[53] Stoit AM, van Schie HT, Slaats-Willemse DI, Buitelaar JK (2013). Grasping motor impairments in autism: not action planning but movement execution is deficient. J Autism Dev Disord.

[54] von der Lühe T, Manera V, Barisic I, Becchio C, Vogeley K, Schilbach L (2016). Interpersonal predictive coding, not action perception, is impaired in autism. Phil Trans R Soc B Biol Sci.

[55] Sebanz N, Knoblich G, Stumpf L, Prinz W (2005). Far from action-blind: representation of others’ actions in individuals with autism. Cog Neuropsychol.

[56] Gowen E, Stanley J, Miall RC (2008). Movement interference in autism-spectrum disorder. Neuropsychologia.

[57] Poliakoff E, Galpin A, Dick J, Moore P, Tipper SP (2007). The effect of viewing graspable objects and actions in Parkinson’s disease. Neuroreport.

[58] Bird G, Leighton J, Press C, Heyes C (2007). Intact automatic imitation of human and robot actions in autism spectrum disorders. Proc R Soc Lond B Biol Sci.

[59] Press C, Richardson D, Bird G (2010). Intact imitation of emotional facial actions in autism spectrum conditions. Neuropsychologia.

[60] Spengler S, Bird G, Brass M (2010). Hyperimitation of actions is related to reduced understanding of others’ minds in autism spectrum conditions. Biol Psych.

[61] Happé F, Ronald A, Plomin R (2006). Time to give up on a single explanation for autism. Nat Neurosci.

[62] Solomon M, Ozonoff SJ, Cummings N, Carter CS (2008). Cognitive control in autism spectrum disorders. Int J Dev Neurosci.

[63] Geurts HM, Corbett B, Solomon M (2009). The paradox of cognitive flexibility in autism. Trends Cogn Sci.

[64] Cook J (2016). From movement kinematics to social cognition: the case of autism. Phil Trans R Soc B.

[65] Mari M, Castiello U, Marks D, Marraffa C, Prior M (2003). The reach-to-grasp movement in children with autism spectrum disorder. Philos TransR Soc Lond B Biol Sci.

[66] Sacrey LR, Germani T, Bryson SE, Zwaigenbaum L (2014). Reaching and grasping in autism spectrum disorder: a review of recent literature. Front Neurol.

[67] Stoit AMB, Schie HT, van Slaats-Willemse DIE, Buitelaar JK (2013). Grasping motor impairments in autism: not action planning but movement execution is deficient. J Autism Dev Disord.

[68] Yang H-C, Lee I-C, Lee I-C (2014). Visual feedback and target size effects on reach-to-grasp tasks in children with autism. J Autism Dev Disord.

