# Supplementary material for: Autistic traits affect interpersonal motor coordination by modulating strategic use of role-based behavior
Source: Mol Autism. 2017 Jun 9;8:23. doi: 10.1186/s13229-017-0141-0 (PMC5466762; doi:10.1186/s13229-017-0141-0)
Supplement: Additional file 1: Table S1. — Individual ADOS and ADI scores. Table S2. Individual Intelligence scales scores. (DOCX 46.5 kb) [file 13229_2017_141_MOESM1_ESM.docx]

**SUPPLEMENTARY RESULTS**

**Table S1: Individual ADOS and ADI scores**

| Participant | ADOS  Social Interaction and Communication |  | ADI  Social Interaction | ADI  Communication | ADI  Restricted  Repetitive  Behavior | ADI  Developmental Abnormalities |
| --- | --- | --- | --- | --- | --- | --- |
| 1 | **12** |  | **8** | **9** | **3** | **0** |
| 2 | **17** |  | **1** | **9** | **3** | **4** |
| 3 | **9** |  | **n.a.** | **n.a.** | **n.a.** | **n.a.** |
| 4 | **13** |  | **n.a.** | **n.a.** | **n.a.** | **n.a.** |
| 5 | **16** |  | **13** | **14** | **3** | **3** |
| 6 | **17** |  | **10** | **12** | **7** | **3** |
| 7 | **8** |  | **8** | **10** | **3** | **3** |
| 8 | **7** |  | **8** | **12** | **1** | **4** |
| 9 | **16** |  | **8** | **15** | **9** | **3** |
| 10 | **16** |  | **n.a.** | **n.a.** | **n.a.** | **n.a.** |
| 11 | **7** |  | **n.a.** | **n.a.** | **n.a.** | **3** |
| 12 | **9** |  | **13** | **10** | **7** | **3** |
| 13 | **13** |  | **n.a.** | **n.a.** | **n.a.** | **2** |
| 14 | **5** |  | **8** | **11** | **9** | **3** |
| 15 | **13** |  | **14** | **12** | **6** | **1** |
| 16 | **15** |  | **14** | **16** | **8** | **2** |

**Table S2: Individual Intelligence scales scores**

| ASC | WAIS-R full scale |  | RAVEN  matrices score | NT | WAIS-R full scale | | RAVEN matrices score |
| --- | --- | --- | --- | --- | --- | --- | --- |
| 1 | 75 |  | - | 1 | - | 100 | |
| 2 | 83 |  | - | 2 | - | 97 | |
| 3 | 94 |  | - | 3 | - | - | |
| 4 | - |  | 100 | 4 | - | 92 | |
| 5 | - |  | 94 | 5 | - | 100 | |
| 6 | - |  | 94 | 6 | - | 97 | |
| 7 | - |  | 100 | 7 | - | 90 | |
| 8 | - |  | 79 | 8 | - | 100 | |
| 9 | - |  | 98 | 9 | - | 94 | |
| 10 | - |  | 100 | 10 | - | 94 | |
| 11 | - |  | 99 | 11 | - | 100 | |
| 12 | - |  | 100 | 12 | - | 84 | |
| 13 | - |  | 96 | 13 | - | 96 | |
| 14 | - |  | 38 | 14 | - | 100 | |
| 15 | - |  | 90 | 15 | - | 100 | |
| 16 | - |  | - | 16 | - | 95 | |

**Data Analysis**

The main text reports parametric ANOVAs with AQ as a continuous predictor also for RT, a variable that, both in the Social Interactive Task and in the Non-Social Interactive Task, deviates from normality. Here we report nonparametric analyses on RT.

**RT** in the **Social Interactive Task**, according to Shapiro-Wilk’s test, is not normally distributed in one (Instructed Opposite Up, Shapiro-Wilk p = 0.004) out of eight conditions and absolute SME-standardized Z-Skewness and Z-Kurtosis for that condition are not normal (>1.96). Nonparametric Friedman ANOVA was significant (Chi Sqr. (32,7) = 108.82, p <0.001). Wilcoxon Matched Pairs Tests, run to investigate main effects of Role and Congruency, showed that when participants played Adaptive (Median = 0.64) had slower RT than when they played Instructed (Median = -0.32) (z=4.92, p = 0.001), and that in Opposite trials (Median = 0.51) participants have slower RT then in Same trials (Median = -0.10) (z= 3.27, p = 0.000001). Both significance levels resist after Bonferroni’s correction for multiple comparisons.

We run nonparametric correlation analysis to investigate further the relation between number of autistic traits (AQ) and RT modulation according to interactive Role or Congruency. Spearman correlation showed that the lower the number of autistic traits (AQ), the longer the RT in the Adaptive, as compared to the Instructed, condition (i.e. indexing more strategic modulation of one’s RTs) (rs = -0.37, p = 0.04). We also investigated the correlation between individual mean RTs and the AQ moderator, separately for the Adaptive and the Instructed conditions. In the Adaptive condition, we found a negative linear correlation (rs = - 0.35, p < 0.05) indicating that the higher the autistic traits, the shorter the time to initiate the movement (i.e. less strategic adaptation). In the Instructed condition, we found a positive correlation (rs = 0.35, p < 0.05) indicating that the higher the autistic traits the longer the time to initiate the movement.

Spearman correlation showed that the higher the autistic traits (AQ), the slower the RT in Opposite compared to Same trials (RT Opposite – RT Same) (rs = 0.41, p = 0.02). We also investigated the correlation between individual mean RTs and the AQ separately for Opposite and Same trials. Correlational analysis showed that for Opposite trials participants with higher autistic traits tended to show longer RT (rs = 0.35, p < 0.05) while for Same trials there was no significant correlation (rs = - 0.28, p = 0.12).

**RT** in the **Non-Social Task**, according to Shapiro Wilks’ test, is not normally distributed in five out of eight conditions (Ps<0.04) and in four of those Skewness is not normal (absolute SME-standardized Skewness > 1.96). Nonparametric Friedman ANOVA was significant (Chi Sqr. (32,7) = 135.67, p <0.001). Wilcoxon Matched Pairs Tests, run in order to investigate Role x Congruency interaction, showed longer RTs in Opposite trials, vs. Same, when taking the Adaptive (Ps < 0.0006) but not the Instructed (Ps > 0.41) role. All significant Ps resist after Bonferroni’s correction for multiple comparisons (for 4 comparisons, corrected α = 0.05/4 = 0.013, for 16 comparisons, corrected α = 0.05/16 = 0.003).

Furthermore, we run nonparametric Spearman correlation analyses in order to investigate whether, also in the Non-Social task, AQ is linked to the change in RT from Adaptive to Instructed condition or from Opposite to Same trials. All correlations were not significant (rs range = -0.16; 0.16, Ps> 0.38). The absence of significant correlations with the AQ indicates that autistic traits were not linked to participants’ RTs in the Non-Social coordination task.

**Between group analyses**

Variables that verified homogeneity of variance (Brown-Forsythe tests) and did not deviate significantly from normality (Shapiro-Wilk’s test and/or normal Skewness and Kurtosis) entered a parametric mixed-model ANOVA with 2 Role (Instructed/Adaptive) x 2 Congruency (Opposite/Same) x 2 Position (Down/Up) as within subjects factors and 2 Group (ASC/NT) as between subjects factor. Variables that did not meet the aforementioned criteria entered nonparametric Friedman ANOVA and Wilcoxon Matched Pairs test.

All tests of significance were based upon an α level of 0.05. Significant interactions and main effects were further analysed by Newman–Keuls post hoc tests.

- We report kinematic measures descriptive of reach-to-grasp trajectory (Maximum Wrist Height = MaxH) and hand’s pre-shaping (Maximum Hand Aperture = MaxAp). These measures were previously shown to describe predictive and adaptive strategies recruited by neurotypical participants during joint grasping task [11, 18]. In particular, when two neurotypical individuals interact, the one playing Instructed facilitates coordination by altering his/her wrist trajectory and grip aperture to signal his/her upcoming movements to his/her partner. On the other hand, participants playing Adaptive are interfered by their partner’s observed opposite movements.
- ***Both in the Social Interactive and in the Non-Social tasks:***
- **Reaction Time** (*ms*) **(RT)**: trial by trial interval between auditory instruction delivery time and start button release time.
- **Movement Duration** (*ms*) **(MD)**: trial by trial interval between start button release time and grasp time.
- **Maximum wrist height** (*mm*) (**MaxH**): trial by trial maximum peak of wrist vertical height during the reach-to-grasp movement.
- **Standard Deviation of Maximum wrist height** (**MaxH-SD**): standard deviation of individual MaxH across trials, an index of between trial variability in wrist trajectory.
- **Maximum Grip Aperture (***mm***) (MaxAp)**: trial by trial maximum peak of the index-thumb 3D Euclidean distance, an index of the grasping component of the reach-to-grasp movement.
- **Standard Deviation of Maximum Grip Aperture (MaxAp-SD)**: standard deviation of individual MaxAp across trials, an index of between trial variability in grasping.

***Only in the Non-Social Task*** :

- **Individual Accuracy (ACC)**: percentage of trials in which each participant grasped the object in the correct location.
- **Grasping Asynchrony** (*ms*) (**GraspAsynch**): trial by trial absolute time interval between participant’s grasp time and dot’s stop time [abs(Participant_grasp-time – Dot_stop-time)].

Pair Accuracy and Grasping Asynchrony in the Social Interactive Task could not be analyzed via between group ANOVA since pairs are composed by one ASC and one NT individuals.

**Social Interactive Task**

**Results**

**Behavioural Measures**

The analysis on **Reaction Times** showed a significant main effect of Role (*F* (1, 30) = 10.11, *p* = 0.003, η^2^ = 0.25), and a significant Role x Group interaction (*F* (1, 30) = 4.78, *p* = 0.03, η^2^ = 0.14). Post hoc tests revealed that in the Adaptive condition NT showed slower RT than ASC(*p* =0.03), and also compared to themselves in the Instructed condition ( *p* < 0.001). In agreement with the moderation analysis, we show that NT participants have a longer RT when they did not know where to grasp the bottle, while this was not the case for ASC participants who showed no modulation of their RTs according to their role (Post hoc test ASC-Instructed vs. ASC-Adaptive: *p* = 0.14).

Results also included significant main effect of Position (*F* (1, 30) = 39.81, *p* < 0.001, η^2^ = 0.57) and of Congruency (*F* (1, 30) = 70.13, *p* < 0.001, η^2^ = 0.70), as well as Role x Congruency (*F* (1, 30) = 30.51, *p* < 0.001, η^2^ = 0.50), Role x Position (*F* (1, 30) = 65.8, *p* < 0.001, η^2^ = 0.68), Congruency x Position (*F* (1, 30) = 27.22, *p* < 0.001, η^2^ = 0.47) and Role x Congruency x Position (*F* (1, 30) = 22.5, *p* < 0.001, η^2^ = 0.42) significant interactions. The latter interaction indicates that participants playing Adaptive have longer RT in Opposite-Up trials. All other *p*s > 0.11.

The analysis on **Movement Duration** showed a significant main effect of Position (*F* (1, 30) = 6.63, *p* = 0.01, η^2^ = 0.18) and a significant Role x Congruency (*F* (1, 30) = 27.69, *p* < 0.001, η^2^ = 0.48) interaction, indicating that participants when playing Instructed vs. Adaptive, showed longer movement duration in Opposite trials (p < 0.001). The Role x Group interaction shows a trend towards significance (*p* = 0.07). All other *p*s > 0.07.

**Kinematics Measures**

The analysis on **MaxH** showed a significant main effect of Position (*F* (1, 30) = 247.70, *p* < 0.001, η^2^ = 0.90), which is explained by the task set up, i.e. higher wrist height is necessary to grasp the bottom (Down) vs. the top (Up) part of the bottle. This effect was modulated by the factor Role (Role x Position interaction = *F* (1, 30) = 41.54, *p* < 0.001, η^2^ = 0.58), showing that when playing Instructed, vs. Adaptive, participants took a lower trajectory when grasping the bottom part of the bottle (*p* < 0.001), and a higher trajectory when grasping the top part of the bottle (*p* < 0.001). The analysis also showed a Role x Congruency significant interaction (*F* (1, 30) = 7.22, *p* =0.01, η^2^ = 0.19), indicating that when playing Adaptive, participants showed a lower trajectory in Same trials compared to all other conditions (*p*s < 0.03).

Analysis on **MaxH-SD** showed that for the ASC group, repeated measure nonparametric Friedman ANOVA was significant (Chi Sqr. (16,7) = 43.25, p < 0.001). Planned Wilcoxon Matched Pairs test for interference effect show that when ASC participants play either Adaptive or Instructed and have to grasp Down their wrist height variability is the same for Opposite and Same trials (Ps> 0.07). For the NT group, repeated measure nonparametric Friedman ANOVA was significant (Chi Sqr. (16,7) = 40.00, p < 0.001). Planned Wilcoxon Matched Pairs test for interference show that there is an interference effect. While when participants play Instructed and have to grasp Down, there is no difference in their wrist height variability depending on their partner movement (p = 1), when they play Adaptive and have to grasp Down, they show greater wrist height variability when they observe their partner grasping Up rather than Down (p = 0.04). This contrast, however, does not survive Bonferroni correction for multiple comparisons (corrected α = 0.05/2 = 0.03).

Analysis on **MaxAp** showed that for the ASC group, repeated measure nonparametric Friedman ANOVA was significant (Chi Sqr. (16,7) = 68.47, p < 0.001). Planned Wilcoxon Matched Pairs test for the interference effect show that, when ASC participants play either Instructed or Adaptive and grasp Up, they do not change their Maximum Grip Aperture depending on where their partner is grasping (Ps>0.46). Therefore, they show no interference effect on their Max Grip Aperture. Planned Wilcoxon Matched Pairs tests show no signalling effect (Ps>0.32).

For the NT group, repeated measure nonparametric Friedman ANOVA was significant (Chi Sqr. (16,7) = 83.94, p < 0.001). Planned Wilcoxon Matched Pairs test for the interference effect show that, when NT participants play Instructed and have to grasp Up, they do not change their Maximum Grip Aperture depending on where their partner is grasping (p = 0.80). Whereas, when they play Adaptive and have to grasp the top, narrow portion of the object, their Max Grip Aperture is larger if they observe their partner grasping the wide, bottom part rather than the narrow top part of the object (Median NT Adaptive Up Opposite = 118.46, Median NT Adaptive Up Same = 114.12, z= 2.17, p = 0.03, r = 0.54). This contrast survives Bonferroni correction for multiple comparisons (corrected α = 0.05/2 = 0.03). Planned Wilcoxon Matched Pairs test show that NT do not signal, in fact they either show no difference between Instructed and Adaptive (Instructed Same Down = Adaptive Same Down, p = 0.13) or show the opposite result i.e., when they grasp down they open their hand more when playing Adaptive than Instructed (Median NT Adaptive Same Down = 132.19, Median NT Instructed Same Down = 129.54, z= 2.17, p= 0.03, r = 0.54). This contrast survives Bonferroni correction for multiple comparisons (corrected α = 0.05/2 = 0.03).

In order to test whether there is a between group difference in the degree of interference effect, we run Mann-Whitney U test to compare the two groups in the contrast, which due to the experimental set up, is more sensitive to show interference on Max Grip Aperture. Results show no between group difference (ASC Adaptive Up (Opposite-Same) median = 2.15, NT Adaptive Up (Opposite-Same) median = 2.57, z = -0.83, p = 0.41, r = -0.15).
There is no between group difference in the degree of signalling (median Instructed Down Average(Opposite, Same) – Adaptive Down Average(Opposite, Same), ASC= 0.16, median NT = - 2.98, Mann-Whitney U test, U = 85, z = 1.62, p = 0.11, r = 0.29).

The analysis on **MaxAP-SD** showed that for the ASC group, repeated measure nonparametric Friedman ANOVA was significant (Chi Sqr. (16,7) = 32.88, p < 0.001). Planned Wilcoxon Matched Pairs test show no significant difference in MaxAp-SD between conditions matched for all characteristics but congruency (Ps> 0.74). For the NT group, repeated measure nonparametric Friedman ANOVA was significant (Chi Sqr. (16,7) = 35.69, p < 0.001). Planned Wilcoxon Matched Pairs test show no significant difference in MaxAp-SD between conditions matched for all characteristics but congruency (Ps> 0.74). Therefore, for both groups, movement variability does not vary as a function of Role and Congruency.

**Non-Social Task**

**Results**

**Behavioural Measures**

The Analysis on **ACC** showed that repeated measure nonparametric Friedman ANOVAs were not significant for either group (ASC: Chi Sqr. (16,7) = 8.88, p = 0.26; NT: Chi Sqr. (16,7) = 9.10, p = 0.25), that is, in each group, participants show similar level of performance in all conditions. Furthermore, results from nonparametric between group Mann-Whitney U tests show that the two groups are similarly accurate in all conditions (Ps>0.50).

The analysis on **Grasping Asynchrony** showed a significant main effect of Group (*F* (1, 30) = 6.63, *p* = 0.01, η^2^ = 0.18), indicating that ASC participants were worse than NT to coordinate with the dot, a significant main effect of Role (*F* (1, 30) = 14.61, *p* < 0.001, η^2^ = 0.32), indicating that participants were more synchronous with the dot when playing Instructed vs. Adaptive, and a significant main effect of Position (*F* (1, 30) = 15.14, *p* < 0.001, η2 = 0.33), indicating that participants were more synchronous with the dot when grasping the Down vs. Up. Results also included Role x Congruency (*F* (1, 30) = 9.00, *p* = 0.005, η^2^ = 0.23), Congruency x Group (*F* (1, 30) = 5.10, *p* = 0.03, η^2^ = 0.14), and Role x Congruency x Group (*F* (1, 30) = 7.26, *p* = 0.01, η^2^ = 0.19) significant interactions. The latter interaction showed that when ASC, but not NT, played Adaptive, they were worse in synchronizing with the dot in Opposite vs. Same trials (*p* < 0.001; NT: *p* = 0.39). Such result is in line with the moderation analysis results , that is, the higher the number of autistic traits the worse participants synchronize with the dot in the Adaptive-Opposite condition. All other *p*s > 0.10.

The analysis on **Reaction Times** showed a significant main effect of Role (*F* (1, 30) = 73.78, *p* <0.001, η^2^ = .71), indicating that participants in the Adaptive condition showed slower reaction times, and a significant main effect of Congruency (*F* (1, 30) = 34.28, *p* <0.001, η^2^ = .53), indicating that participants showed slower reaction times in the Opposite trials.

Results also included a Role x Congruency (*F* (1, 30) = 25.91, *p* < 0.001, η^2^ = 0.46), indicating that when participants –played Adaptive they showed slower reaction times in Opposite trials, and a Congruency x Position (*F* (1, 30) = 17.0, *p* = 0.0002, η^2^ = 0.36) significant interactions, indicating that when participants played Adaptive (vs. Instructed), they showed longer reaction times in both Up and Down. All other *p*s > 0.05. Absence of significant interactions with the factor Group corroborates the results of the moderation analysis, indicating that autistic traits did not modulate participants’ reaction times in the individual coordination task.

The analysis on **Movement Duration** showed main effects of Position (*F* (1, 30) = 76.31, *p* < 0.001, η^2^ = 0.71), showing longer movements when grasping the bottle in the Up position, Congruency (*F* (1, 30) = 10.46, *p* = 0.002, η^2^ = 0.25), indicating longer movements in the Opposite trials, and a Congruency x Position significant interaction (*F* (1, 30) = 5.38, *p* = 0.02, η^2^ = 0.15), showing longer movements in Opposite vs. Same trials both in Up and Down grasping (*p*s <0.001). All other *p*s > 0.06.

**Kinematics Measures**

The analysis on **MaxH** showed main effects of Role (F (1, 30) = 6.76, *p* = 0.01, η^2^ = 0.18), Congruency (F (1, 30) = 6.47, *p* = 0.01, η^2^ = 0.17), and Position (F (1, 30) = 1878,19, *p* < 0.001, η^2^ = 0.98), as well as Role x Congruency (F (1, 30) = 11.58, *p* = 0.002, η^2^ = 0.28), Role x Position (F (1, 30) = 63.73, *p* < 0.001, η^2^ = 0.67), Congruency x Position (F (1, 30) = 23.63, *p* <0.001, η^2^ = 0.44), and Role x Congruency x Position significant interactions (F (1, 30) = 14.24, *p* < 0.001, η^2^ = 0.32). The latter interaction indicated that when participants played Adaptive, they performed movements with larger maximum wrist height (MaxH) in Opposite-Down trials, compared to when they played Instructed (*p* < 0.001), and compared to Same trials (p <0.001). All other *p*s > 0.07.

The analysis on **MaxH-SD** showed that for the ASC group, repeated measure nonparametric Friedman ANOVA was significant (Chi Sqr. (16,7) = 44.13 p < 0.001). ASC participants show greater movement variability in Opposite vs Same trials only when they play Adaptive (Planned Wilcoxon Matched Pairs, z=3.52, p=0.0004, r= 0.88) and not when they play Instructed (Wilcoxon Matched Pairs, z=0.10, p=0.92). Significance survives after Bonferroni correction for multiple comparisons (corrected α = 0.05/2 = 0.03).
For the NT group, repeated measure nonparametric Friedman ANOVA was significant (Chi Sqr. (16,7) = 50.50 p < 0.001). As expected, NT participants show greater movement variability in Opposite vs Same trials only when they play Adaptive (Planned Wilcoxon Matched Pairs, z=3.21, p=0.001, r= 0.80) and not when they play Instructed (Wilcoxon Matched Pairs, z=0.78, p=0.44). Significance survives after Bonferroni correction for multiple comparisons (corrected α = 0.05/2 = 0.03). We run Mann-Whitney U test to test whether there is a between group difference in the degree of interference effect. Results show no group difference (Instructed Down Average(Opposite, Same) – Adaptive Down Average(Opposite, Same), median ASC= 0.16, median NT = - 2.98, Mann-Whitney U test, U = 112, z = 0.60, p = 0.55, r = 0.11).

The analysis on **MaxAp** showed significant main effects of Role (*F* (1, 30) = 5.57, *p <* 0.001, η^2^ = 0.71), Position (*F* (1, 30) = 892.195, *p <* 0.001, η^2^ = 0.96) due to the task set up (i.e. larger grip aperture is necessary when grasping Down compared to Up) and Congruency (*F* (1, 30) = 10.46, *p =* 0.002, η^2^ = .25), indicating larger grip aperture when performing Opposite trials. Results also included a Role x Position significant interaction (*F* (1, 30) =16.32, *p* = 0.0003, η^2^ = 0.35), indicating that participants showed larger grip aperture in Up grasping when playing Adaptive (*p* = 0.001).

The analysis on **MaxAP-SD** showed a significant main effect of Position (*F* (1, 30) = 169.32, *p* < 0.001, η^2^ = 0.84), and significant Role x Position (*F* (1, 30) = 7.14, *p* = 0.01, η^2^ = 0.19), Position x Group (*F* (1, 30) = 4.93, *p* = 0.03, η^2^ = 0.14), and a Role x Congruency x Position (*F* (1, 30) = 6.93, *p* = 0.01, η^2^ = 0.18) significant interactions. When participants played Adaptive they showed higher grasp variability in all conditions compared to Instructed (all *ps* < 0.04). All other *p*s > 0.23.
